# Scotland's First Open-Air Hospital

**Published:** 1916-04-15

**Authors:** Margaret Ker

**Affiliations:** Hon. Treasurer and Secretary.


### "Scotland's First Open-Air Hospital."

Easterton, Milngavie, Scotland.

Sir,—In your issue of 1st inst., Sir Thomas Burnett, of Leys, chairman of the Royal Aberdeen Hospital for Sick Children, is reported to have said that no open-air hospital has been built in Scotland.

I am instructed to point out that this is a mistake, and to send you the last report of the Children's Home Hospital, Strathblane (the twelfth), which, since it came to Strathblane in 1913, has carried out the open-air treatment with great success.

The patients spend practically all their time on the verandah which runs the whole length of the building, and sleep there even in the coldest weather.

I shall be obliged if you will insert this correction in your next number.—I am, yours truly,

Margaret Ker, Hon. Treasurer and Secretary.

